# Supplementary material for: Low-dose self-amplifying mRNA COVID-19 vaccine drives strong protective immunity in non-human primates against SARS-CoV-2 infection
Source: Nat Commun. 2022 Jun 7;13:3289. doi: 10.1038/s41467-022-31005-z (PMC9173840; doi:10.1038/s41467-022-31005-z)
Supplement: Supplementary file 1 — Supplementary Information [file 41467_2022_31005_MOESM1_ESM.pdf]

Supplementary Information

Rappaport et al.

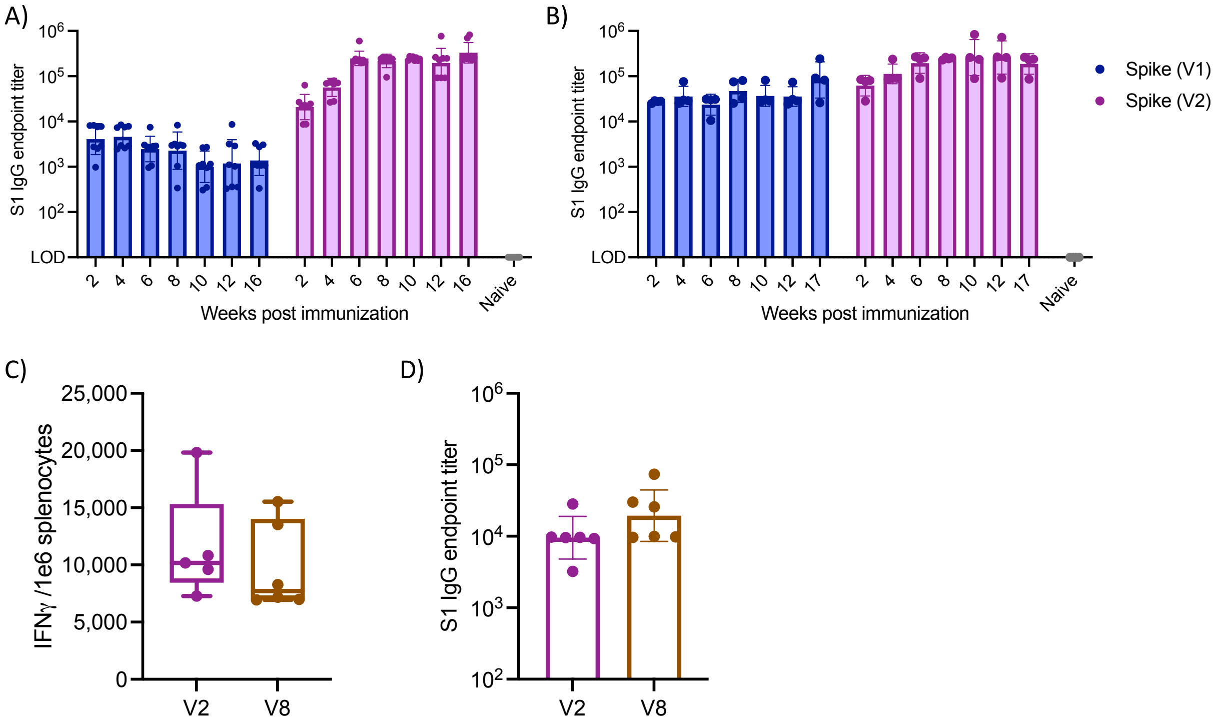

Supplementary Figure 1. Serum spike S1 IgG antibody binding titers in Balb/c mice assessed by MSD ELISA at the specified timepoint post immunization with either (A) ChAd (1×10^{11} VP) or (B) SAM (10 µg) encoding either the Spike V1 or V2 sequence. Naïve samples all below LOD (LOD = 10). Geomean and geometric SD of n = 8 (A) or n = 4 (B) independent animals. (C) Spike-specific T-cell response assessed by IFNγ ELISpot (sum of eight pools) in Balb/c mice. Splenocytes collected 2 weeks post immunization with ChAd (1×10^{9} VP) encoding either the spike V2 or V8 sequence. Box and whiskers represent median, IQR and range; n = 6 independent animals per group, one sample excluded from V2 group due to high variance of technical replicates. (D) Serum spike S1 IgG antibody binding titers in Balb/c mice assessed by MSD ELISA 2 weeks post immunization with ChAd (1×10^{9} VP) encoding either the spike V2 or V8 sequence. Geomean and geometric SD of n = 6 independent animals, one experiment.

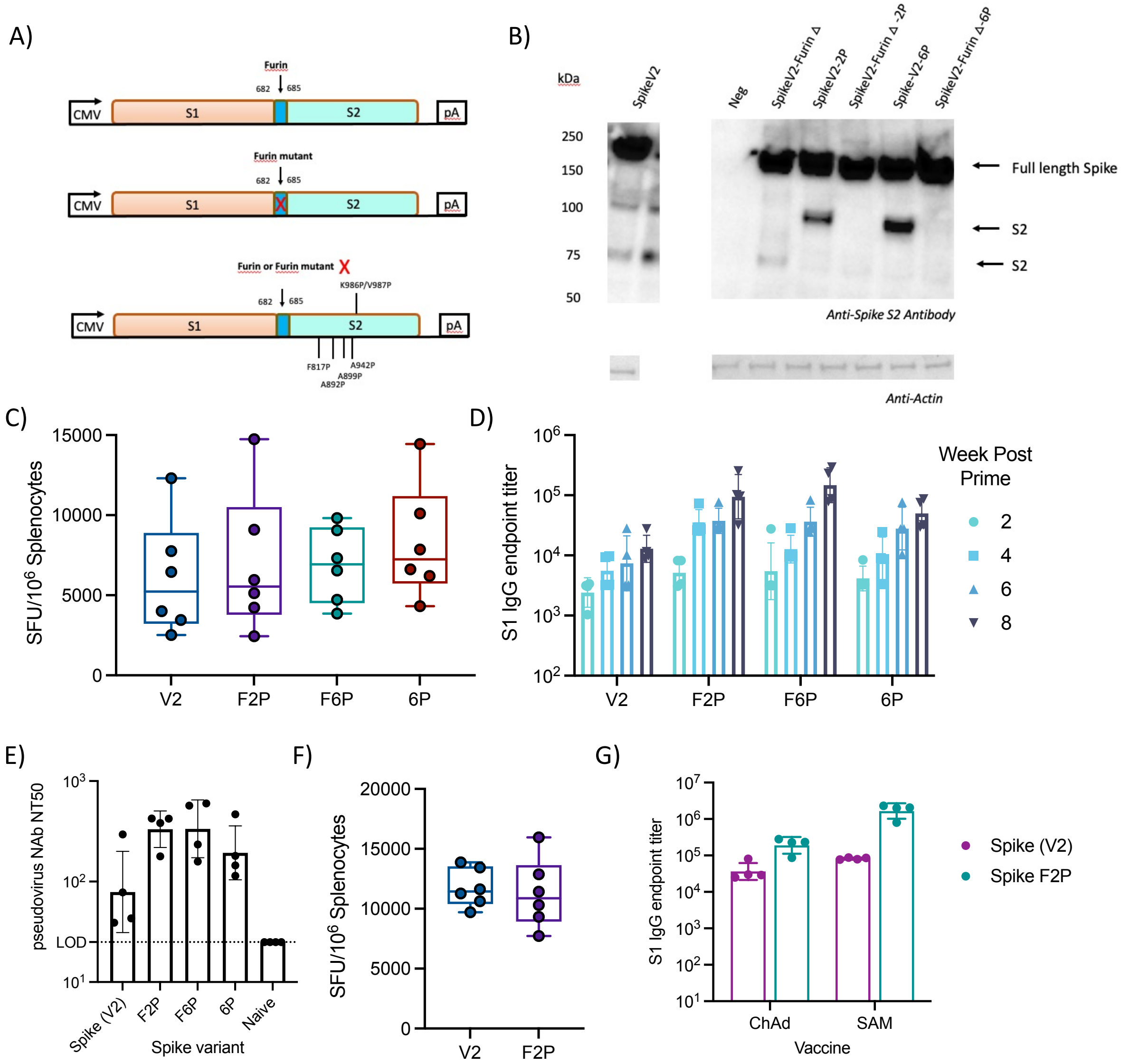

Supplementary Figure 2. Addition of Furin mutation and proline substitutions increases neutralizing antibody titers in mice. (A) Schematic of ChAd constructs expressing a codon optimized spike V2 either as a wild-type protein or with mutations in the Furin cleavage site and/or with proline stabilization mutations: WT-Spike, wild-type spike; FurinΔ Spike, Furin or Furin mutated spike with 2-proline, K986P/V987P (2P) or 6-proline mutations F817P, A892P, A899P, A942P, K986P & V987P (6P). (B) Western blot of HEK293F cell lysates analyzed 72h post infection (MOI 1) with ChAd-SpikeV2 constructs or with a negative control ChAd (Neg) expressing a non-spike protein, using an anti-S2 antibody. Samples were run on a second gel in parallel and an anti-Actin antibody was used to confirm equivalent protein loading for all samples. Experiment performed once. (C) Spike specific T cell response in Balb/c mice

splenocytes 2 weeks post immunization with ChAd vaccine encoding the specified spike variant ($1x10^{10}$ VP), IFNγ ELISpot, sum of eight spike peptide pools. Box represents interquartile range (IQR) (25 – 75%) with median line, and whiskers represent range; n = 6 animals per group over one experiment. (D) Serum spike S1 IgG antibody binding titers in Balb/c mice assessed by MSD ELISA at the specified timepoint post immunization with ChAd vaccine encoding the specified spike variant ($1x10^{10}$ VP). (E) Serum pseudovirus neutralization titers (50% inhibition) in Balb/c mice 4 weeks post immunization with ChAd vaccine ($1x10^{10}$ VP) encoding the specified spike variant. Naïve samples all below LOD of 25. (D & E) Geomean and geometric SD of n = 4 independent animals per group over one experiment. (F) Spike specific T cell response in Balb/c mice splenocytes 2 weeks post immunization with SAM (10 μg) vaccine encoding the specified spike variant, IFNγ ELISpot, sum of two spike peptide pools. Box represents interquartile range (IQR) (25 – 75%) with median line, whiskers represent range; n = 6 independent animals, representative data from one of two experiments. (G) Serum spike S1 IgG antibody binding titers in Balb/c mice assessed by MSD ELISA at 4 weeks post immunization with ChAd ($1x10^{11}$ VP) or SAM (10 μg) vaccine encoding the specified spike variant. Geomean and geometric SD of n = 4 independent animals per group, representative data from one of three experiments.

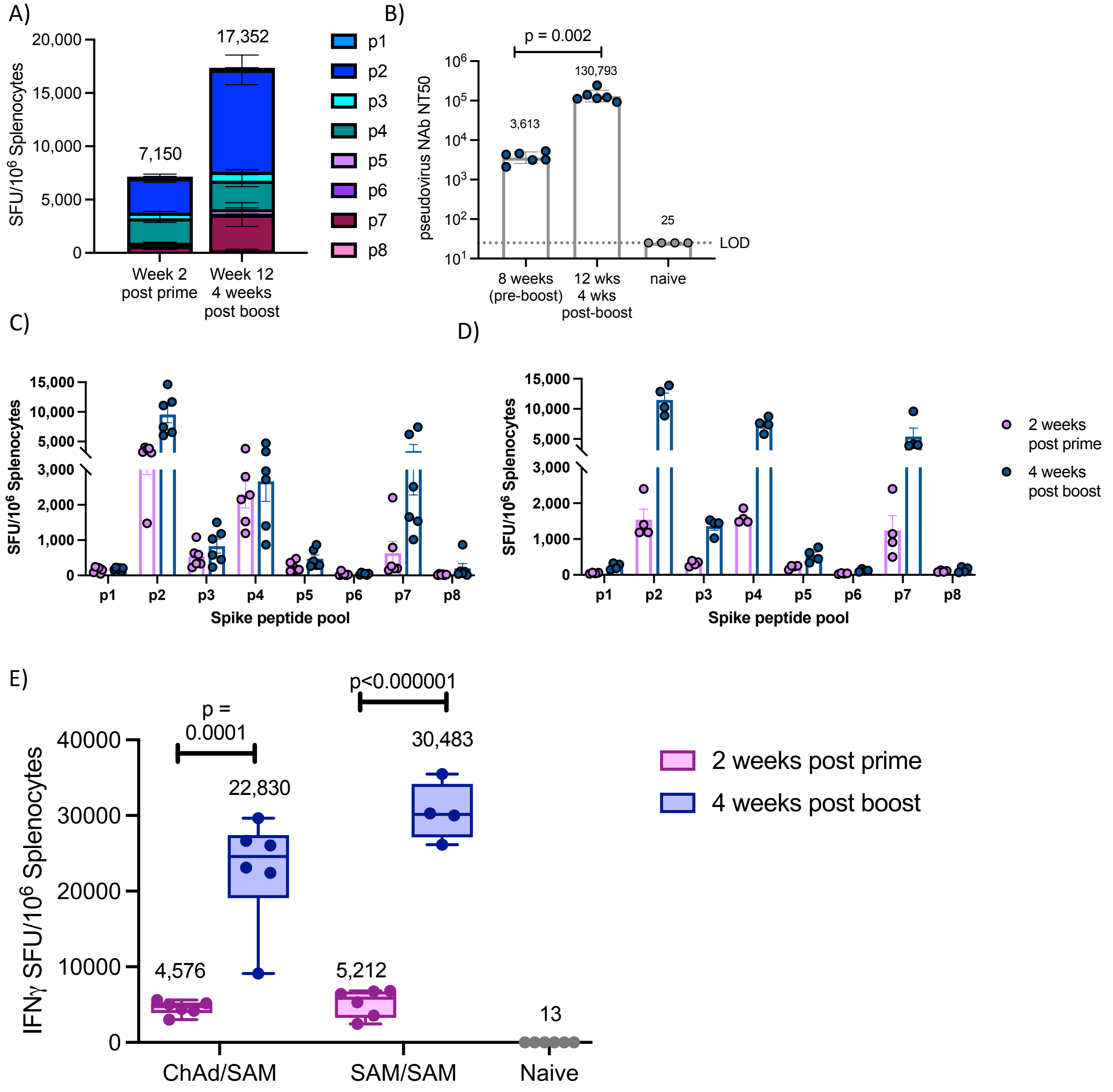

Supplementary Figure 3. Prime/boost in Balb/c mice. (A – C) Mice immunized with ChAd-(V2)F2P ($1x10^{11}$ VP) prime, followed by SAM-(V2)F2P (10 µg) boost 8 weeks post prime. N = 6/group. (A) IFNγ ELISpot at the specified timepoint, following stimulation with eight peptide pools spanning spike antigen. Mean ± SEM for each peptide pool. Note that mice were immunized at the same time and separate cohorts were assessed at the specified timepoints. Data from one of four studies is shown. (B) Pseudovirus neutralizing titer (NT50) at the specified timepoint in the same cohort of mice that were assessed in (A) at 4 weeks post boost. Geometric mean and geometric SD. Sera from Naïve mice were below LOD of 25. Two-tailed Mann-

Whitney test. (C) IFNγ ELISpot values for each peptide pool for individual mice presented in panel (A) Mean +/- SE for n = 6 mice per group. (D) IFNγ ELISpot values for each peptide pool for individual mice presented in main Figure 2A. Mice immunized with SAM-(V2)F2P (10 μg) at week 0 and week 8. Immunizations staggered and splenocytes collected and assessed together at 2 weeks post prime and 4 weeks post boost. Mean +/- SE for n = 4 mice per group. (E) Repeat study with independent mice for experiments shown in main Figure 2 and Supplementary Figure 3. Balb/c mice immunized with ChAd-Spike(V2)F2P ($5x10^{10}$ VP) or SAM-Spike(V2)F2P (10 μg) prime, followed by SAM-Spike(V2)F2P (10 μg) boost at 12 weeks post prime. Separate cohorts received prime immunization at week 12. All mice (including unvaccinated controls) were euthanized and splenocytes collected at week 16 (4 weeks post prime or boost) and assessed at the same time by IFNγ ELISpot. Sum of two peptide pools spanning spike antigen for each animal. N = 6 mice per group, except for SAM-SAM post boost, which was n = 4 mice. Box and whiskers are median, IQR and range. Annotated number is mean. P-values – unpaired Student's t-tests (two-tailed).

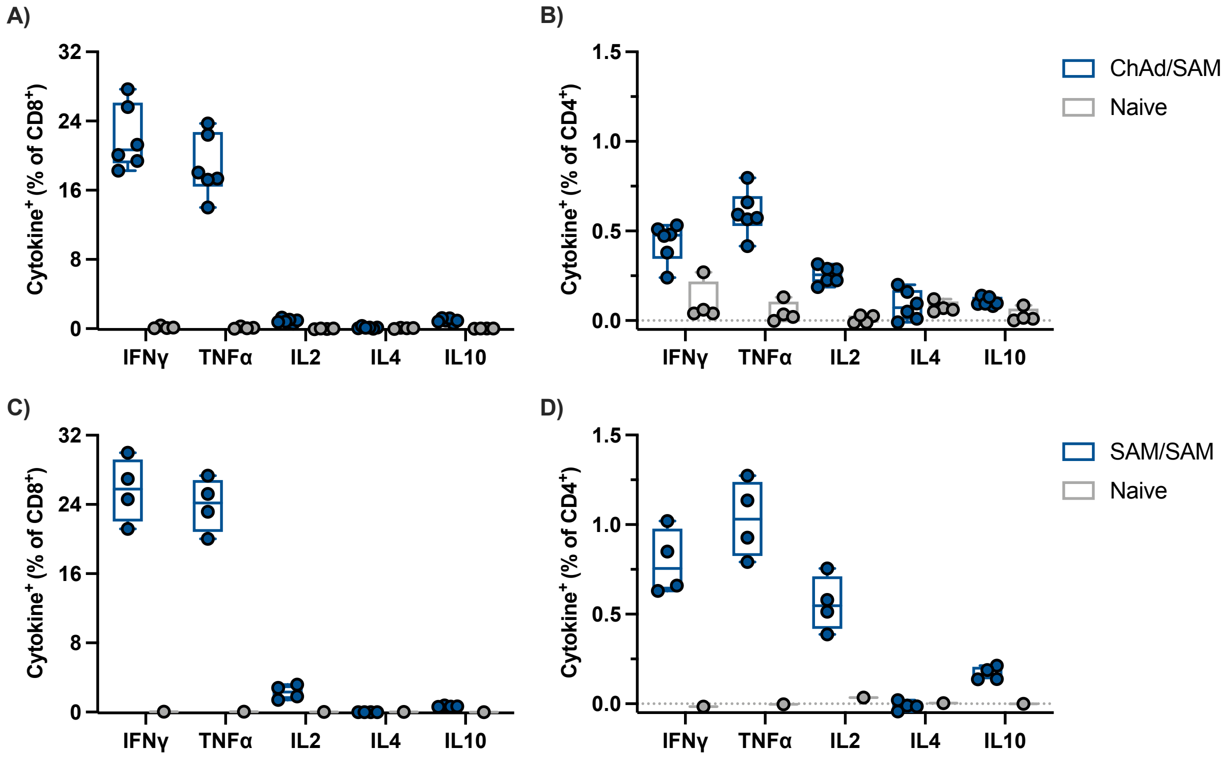

Supplementary Figure 4. Vaccine induced T cell response is T_H1 biased. Intracellular cytokine staining in splenocytes of Balb/c mice following stimulation with overlapping peptide pool spanning spike. Background corrected, box and whiskers represent median, IQR, and range. **(**A-B) Mice immunized with $1x10^{11}$ VP ChAd-Spike(V2)-F2P and boosted with SAM-Spike(V2)-F2P (10 µg) at 8 weeks. T cell response assessed at week 12, 4 weeks post boost (n=6) compared to unvaccinated mice (n=4). **(**C-D) Mice immunized with SAM-Spike(V2)-F2P (10 µg) at 0 and 8 weeks. T cell response assessed at week 10, 2 weeks post boost (n=4) compared to unvaccinated mice (n=1). **(**A&C) Cytokine positive out of CD8+ T cells (%) **(**B&D) Cytokine positive out of CD4+ T cells (%).

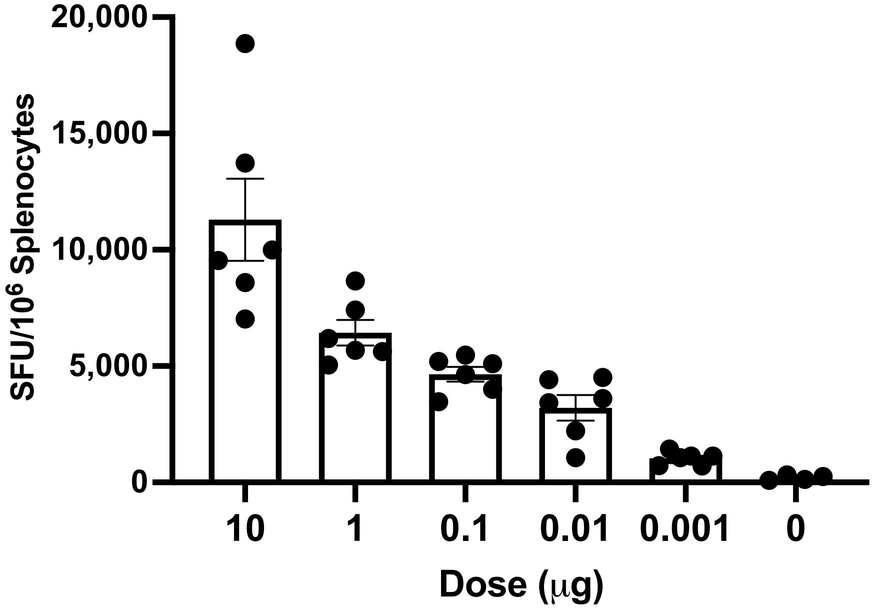

Supplementary Figure 5. SAM induces a dose-dependent T cell response in mice. Antigen-specific T cell response assessed by IFNγ ELISpot (sum of eight spike pools) in splenocytes in Balb/c mice (n=6/group) 2 weeks post immunization with SAM-Spike(V1) at the specified dose. Mean ± SE.

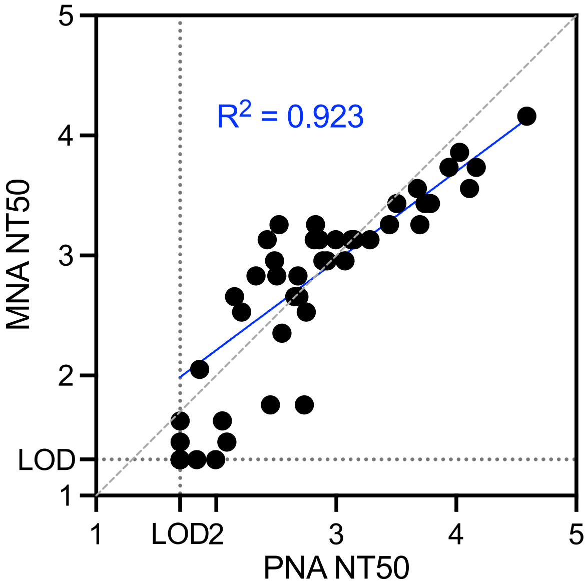

Supplementary Figure 6. Scatterplot comparing NT50 titers measured by pseudovirus neutralization assay (PNA) and live virus microneutralization assay (MNA) in rhesus macaques for all sera samples that were assessed by both assays (excluding baseline samples) at various timepoints post vaccination (n = 25 individual animals, n = 50 total samples). Dashed gray line represents unity. LOD for MNA assay = 20, LOD for PNA assay = 50. Blue line represents non-linear regression analysis best fit.

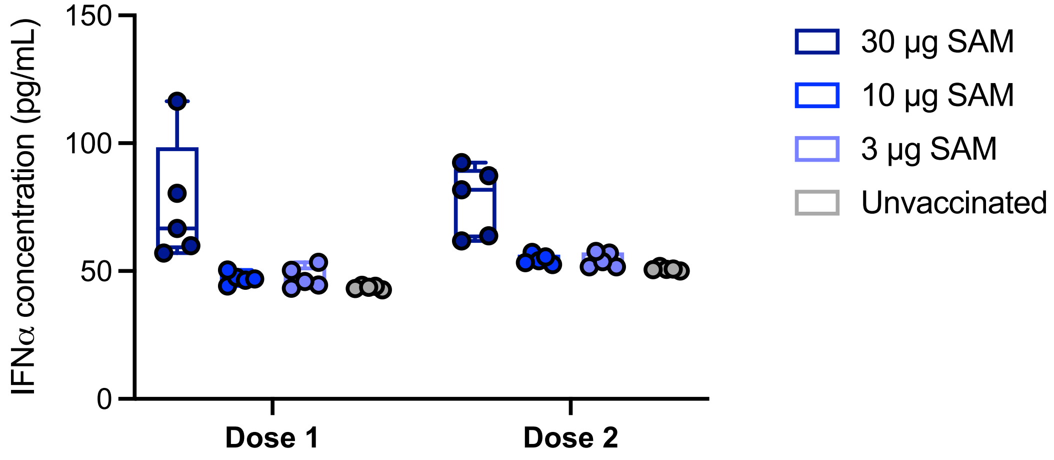

Supplementary Figure 7. Serum IFN α2α concentration assessed by MSD in rhesus macaques (n=5/group) 8 hours post immunization with SAM-Spike(V2)-F2P at the specified dose. Box represents interquartile range with median line, whiskers denote range.

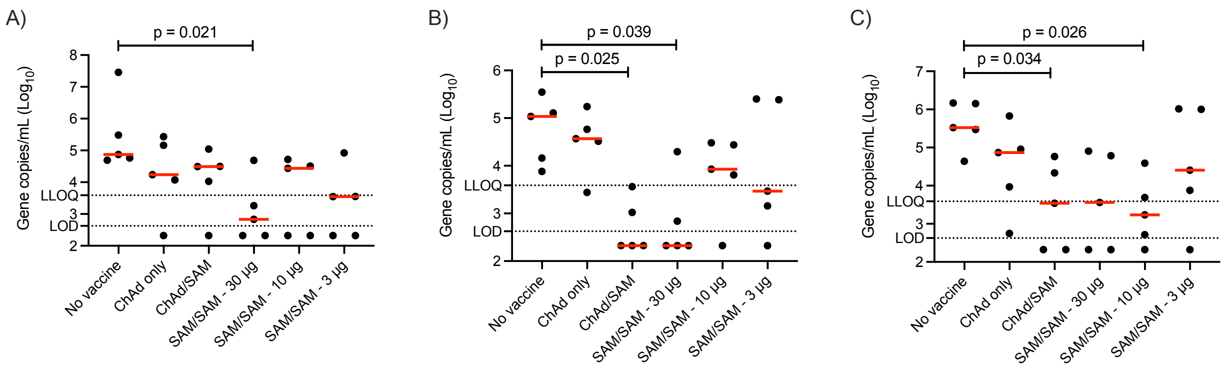

Supplementary Figure 8. Viral replication is reduced in vaccinated NHP following SARS-CoV-2 challenge. (A) Peak subgenomic RNA (sgRNA) levels in bronchial alveolar lavage for each animal determined by RT-qPCR between day 1 and day 10 post SARS-CoV-2 challenge. (B) Peak sgRNA levels in oropharyngeal swab for each animal determined by RT-qPCR between day 1 and day 14 post SARS-CoV-2 challenge. (C) Peak sgRNA levels in nasal swab for each animal determined by RT-qPCR between day 2 and day 14 post SARS-CoV-2 challenge. LOD = 422, samples below LOD set to ½ LOD. LLOQ = 3,881. Red bar is median of n = 5 independent animals per group over one experiment. Statistical analyses: Kruskal-Wallis followed by Dunn's multiple comparison post-test, adjusted p-values shown for comparisons with $p < 0.05$.

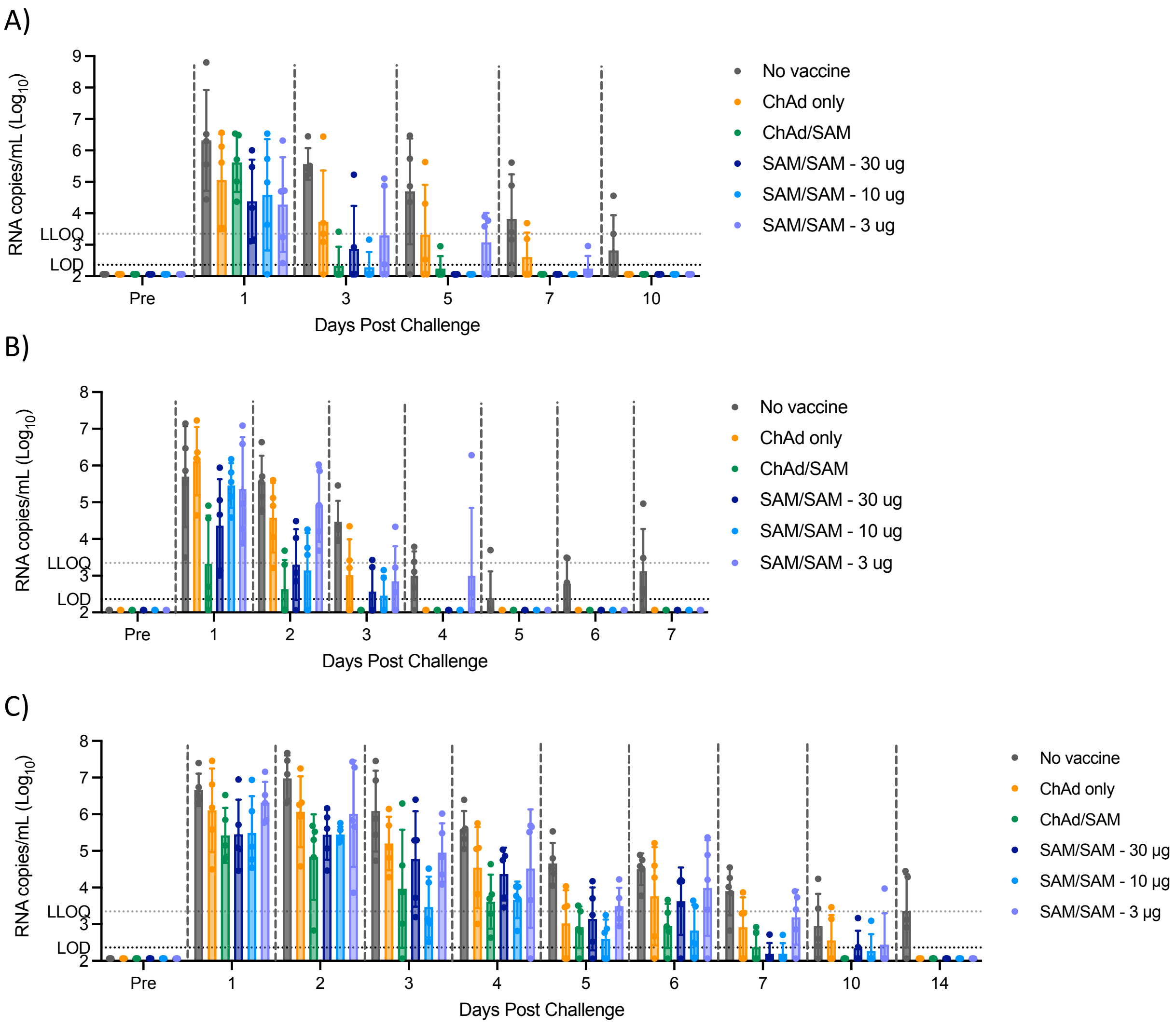

Supplementary Figure 9. Total genomic RNA levels (N1) determined by RT-qPCR at specified timepoint post SARS-CoV-2 challenge for each animal in (A) bronchial alveolar lavage (B) oropharyngeal swab or (C) nasal swab. LOD = 230, samples below LOD set to ½ LOD. Geometric mean and SD of n = 5 independent animals per group over one experiment.

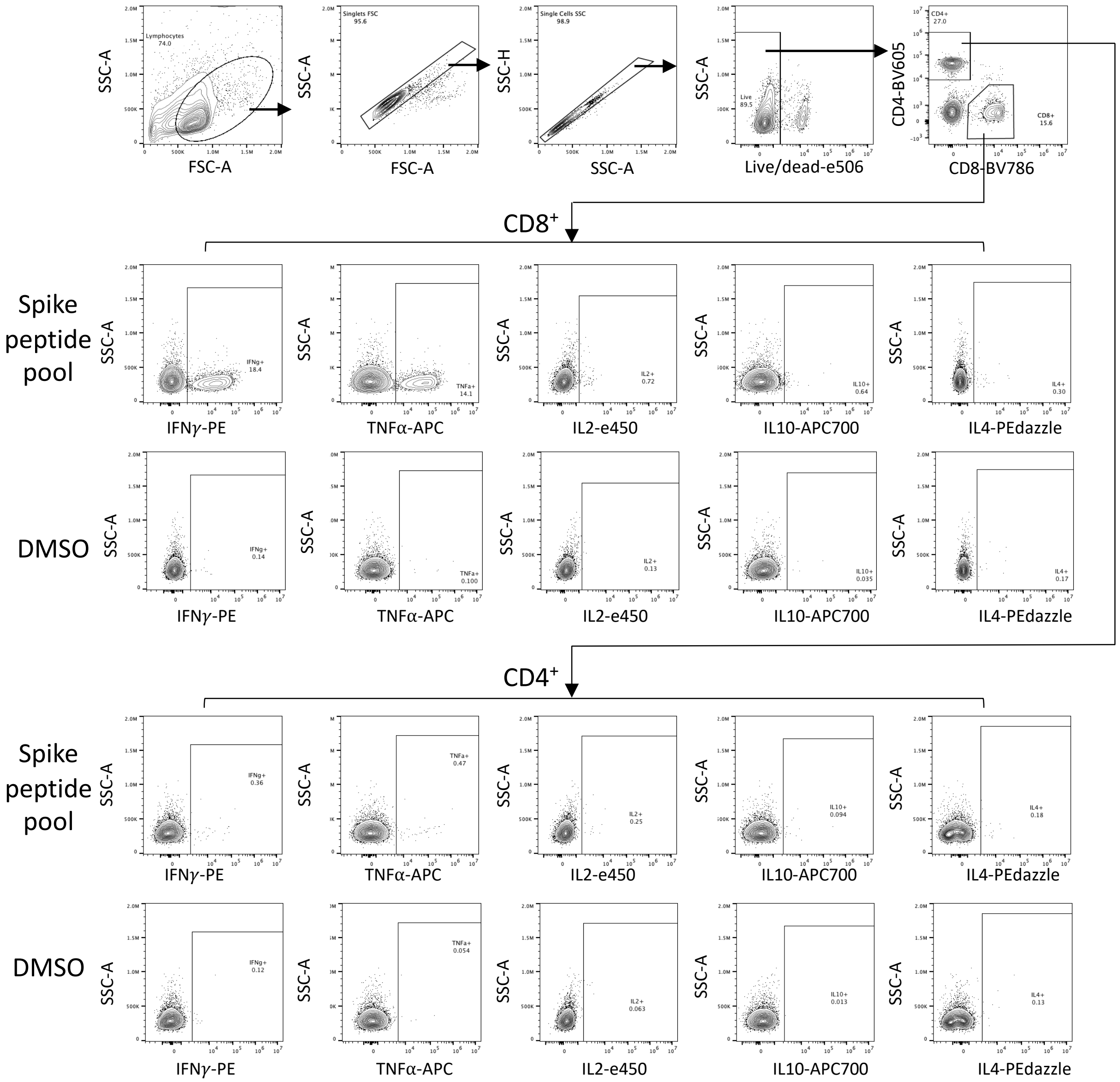

Supplementary Figure 10. Representative gating strategy for intracellular cytokine staining analysis (data shown in Supplementary Fig. 4). Balb/c mouse splenocytes following stimulation with overlapping peptide pool spanning Spike antigen or DMSO control.

Supplementary Table 1. Non-human primates (rhesus macaques) used in study

Animal Number	Subject Name	Group	Treatment	Sex	Challenge Day	DOB	Body Weight (kg) 2/10/2021	Body Weight (kg) 2/25/2021
1	17C130	1	ChAd/SAM	Male	A	5/19/17	7.18	6.91
2	17C324	1		Female	A	9/19/17	6.61	6.4
3	17C037	1		Female	A	4/2/17	5.57	5.5
4	17C167	1		Male	B	6/4/17	5.26	5.33
5	17C123	1		Female	B	5/17/17	3.83	3.8
6	17C045	2	ChAd only	Male	A	4/5/17	6.82	7.02
7	17C260	2		Female	A	7/11/17	5.07	5.2
8	17C227	2		Female	B	6/25/17	6.59	6.4
9	17C155	2		Male	B	5/30/17	5.36	5.59
10	17C313	2		Female	B	8/28/17	4.94	4.8
11	17C126	3	SAM – 30 μg	Male	A	5/18/17	6.87	6.87
12	17C172	3		Female	A	6/4/17	5.93	5.8
13	17C151	3		Female	A	5/30/17	5.21	5.3
14	17C240	3		Male	B	7/3/17	5.89	5.68
15	17C264	3		Female	B	7/13/17	4.9	4.8
16	17C152	4	SAM – 10 μg	Male	A	5/30/17	5.23	5.27
17	17C136	4		Female	A	5/19/17	4.9	5
18	17C192	4		Female	B	6/11/17	5.62	5.6
19	17C002	4		Male	B	1/17/17	7.26	7.2
20	17C058	4		Female	B	4/18/17	4.61	4.6
21	17C329	5	SAM – 3 μg	Male	A	9/27/17	4.97	4.91
22	17C185	5		Female	A	6/9/17	5.13	5.1
23	17C257	5		Female	A	7/10/17	6.62	6.5
24	17C214	5		Male	B	6/19/17	6.17	6.08
25	17C337	5		Female	B	11/9/17	4.77	4.5
26	17C254	6	Control (PBS)	Male	A	7/10/17	5.79	5.88
27	17C072	6		Female	A	4/26/17	6.53	6.6
28	17C220	6		Male	B	6/22/17	8.18	7.94
29	17C092	6		Male	B	5/8/17	6.2	6.19
30	17C056	6		Female	B	4/14/17	5.4	5.3

Western Blot Images

Full uncropped and unprocessed scans for blots in Figure 1A and Supplementary Figure 2B.

Figure 1A

Anti-S2

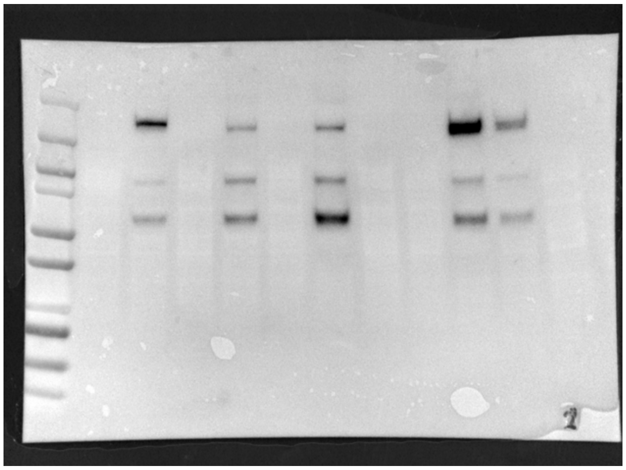

Anti-Actin

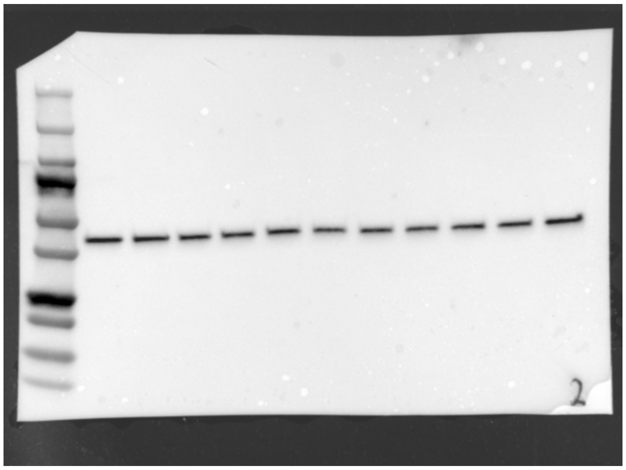

Supplementary Figure 2B

Anti-S2

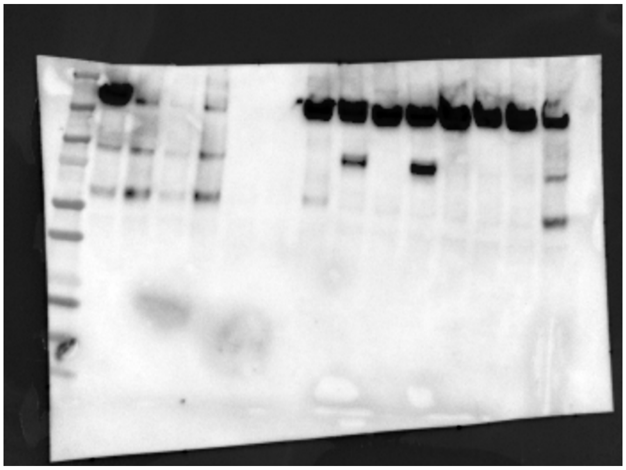

Anti-Actin

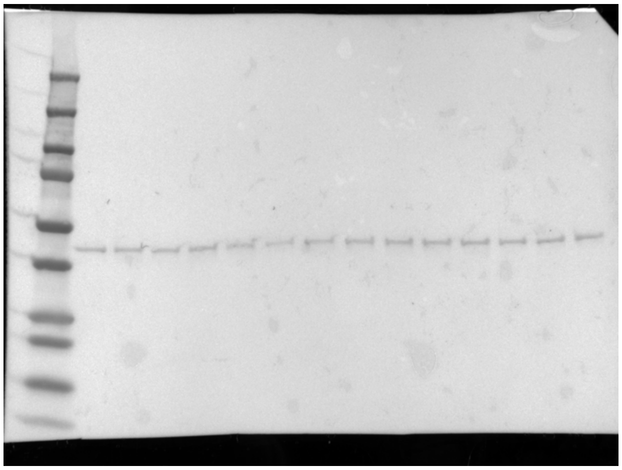

*Note – lanes 2 – 5 are vaccine constructs that are not relevant to the study described in the manuscript.
