# Supplementary material for: Low-dose self-amplifying mRNA COVID-19 vaccine drives strong protective immunity in non-human primates against SARS-CoV-2 infection
Source: Nat Commun. 2022 Jun 7;13:3289. doi: 10.1038/s41467-022-31005-z (PMC9173840; doi:10.1038/s41467-022-31005-z)
Supplement: Supplementary file 3 — Reporting Summary [file 41467_2022_31005_MOESM3_ESM.pdf]

nature portfolio

Corresponding author(s): Karin Jooss

Last updated by author(s): May 1, 2022

Reporting Summary

Nature Portfolio wishes to improve the reproducibility of the work that we publish. This form provides structure for consistency and transparency in reporting. For further information on Nature Portfolio policies, see our Editorial Policies and the Editorial Policy Checklist.

Statistics

For all statistical analyses, confirm that the following items are present in the figure legend, table legend, main text, or Methods section.

n/a	Confirmed	
☐	☒	The exact sample size (*n*) for each experimental group/condition, given as a discrete number and unit of measurement
☐	☒	A statement on whether measurements were taken from distinct samples or whether the same sample was measured repeatedly
☐	☒	The statistical test(s) used AND whether they are one- or two-sided<br>*Only common tests should be described solely by name; describe more complex techniques in the Methods section.*
☒	☐	A description of all covariates tested
☐	☒	A description of any assumptions or corrections, such as tests of normality and adjustment for multiple comparisons
☐	☒	A full description of the statistical parameters including central tendency (e.g. means) or other basic estimates (e.g. regression coefficient) AND variation (e.g. standard deviation) or associated estimates of uncertainty (e.g. confidence intervals)
☐	☒	For null hypothesis testing, the test statistic (e.g. *F*, *t*, *r*) with confidence intervals, effect sizes, degrees of freedom and *P* value noted<br>*Give P values as exact values whenever suitable.*
☒	☐	For Bayesian analysis, information on the choice of priors and Markov chain Monte Carlo settings
☒	☐	For hierarchical and complex designs, identification of the appropriate level for tests and full reporting of outcomes
☒	☐	Estimates of effect sizes (e.g. Cohen's *d*, Pearson's *r*), indicating how they were calculated

Our web collection on statistics for biologists contains articles on many of the points above.

Software and code

Policy information about availability of computer code

Data collection	IgG ELISA and serum cytokine data collected using Methodical Mind software version 1.0.37 (MesoScale Discovery). PNA data collected using SoftMax Pro version 6.5.1. ELISpot was imaged using vSpot v7 (Autoimmun Diagnostika). Codon optimization performed using COOL optimization algorithm version 20160707a and SGI-DNA codon optimization tool version 20190802.
Data analysis	Flow cytometry analysis performed using Flow Jo (version 10.7.1). ELISpot, IgG, and PNA data was analyzed using R version 3.5.3. Graphing and statistical analysis was performed using GraphPad Prism 9.

For manuscripts utilizing custom algorithms or software that are central to the research but not yet described in published literature, software must be made available to editors and reviewers. We strongly encourage code deposition in a community repository (e.g. GitHub). See the Nature Portfolio guidelines for submitting code & software for further information.

Data

Policy information about availability of data

All manuscripts must include a data availability statement. This statement should provide the following information, where applicable:

- Accession codes, unique identifiers, or web links for publicly available datasets
- A description of any restrictions on data availability
- For clinical datasets or third party data, please ensure that the statement adheres to our policy

The data generated in this study are provided in the Source Data file.

Field-specific reporting

Please select the one below that is the best fit for your research. If you are not sure, read the appropriate sections before making your selection.

☒ Life sciences ☐ Behavioural & social sciences ☐ Ecological, evolutionary & environmental sciences

For a reference copy of the document with all sections, see nature.com/documents/nr-reporting-summary-flat.pdf

Life sciences study design

All studies must disclose on these points even when the disclosure is negative.

Sample size	For evaluating disease progression post-challenge as measured by RT-qPCR, MN, ELISA, temperature, body weights, clinical chemistry, and hematology, there will be five animals in each vaccinated group and five animals in the control group. These sample sizes will provide greater than 81% power to detect a 2.6-fold change between each vaccinated dose group and the control group as well as greater than 80% power to detect a 3.3-fold change between any two vaccinated dose groups for any of the continuous parameters that are log-transformed for the analysis. This assumes a 40% coefficient of variation for the continuous parameter. For parameters that are not log-transformed, the sample sizes will provide greater than 80% power to detect a 4.9 standard-unit change between each vaccinated dose group and the control group as well as greater than 80% power to detect a 6.2 standard-unit change between any two vaccinated dose groups assuming a standard deviation of two. These power calculations performed are for one-sided t-tests comparing vaccinated animals to controls and two-sided t-tests comparing any two of the vaccinated groups using a Bonferroni multiple comparison adjustment to control the overall Type I error level at no more than 5% for each set of tests. They were performed using the power procedure in SAS (version 9.4).
Data exclusions	One sample at one timepoint was excluded from ELISpot analysis based on predefined criteria, triplicate well variability was greater 10 and median greater than 10. Two samples excluded from MN analysis based on failure of predefined QC criteria.
Replication	Mouse experiments were repeated at least twice as independent experiments, this is indicated in the figure legends for each experiment. All replicate studies gave similar results to those shown. NHP study was performed once due to limitation of animal model and ethical considerations. NHP samples were run in technical duplicate or triplicates for all assays.
Randomization	NHP and mice were randomized into groups based on animal ID. Allocation was random for all animal studies.
Blinding	Investigators were not blinded during data collection or analysis. Blinding was not relevant as the reported data were not based on subjective observations, but quantitative measurements, including RT-PCR, ELISA, ELISpot and PNA.

Reporting for specific materials, systems and methods

We require information from authors about some types of materials, experimental systems and methods used in many studies. Here, indicate whether each material, system or method listed is relevant to your study. If you are not sure if a list item applies to your research, read the appropriate section before selecting a response.

Materials & experimental systems

n/a	Involved in the study
☐	☒ Antibodies
☐	☒ Eukaryotic cell lines
☒	☐ Palaeontology and archaeology
☐	☒ Animals and other organisms
☒	☐ Human research participants
☒	☐ Clinical data
☒	☐ Dual use research of concern

Methods

n/a	Involved in the study
☒	☐ ChIP-seq
☐	☒ Flow cytometry
☒	☐ MRI-based neuroimaging

Antibodies

Antibodies used	Antibodies for flow cytometry:<br>Rat Anti-Mouse IFNgamma clone XMG1.2, Invitrogen, Cat #: 12-7311-82, PE (1 uL/1e6 cells)<br>Rat Anti-Mouse TNFalpha clone MP6-XT22, eBioscience, Cat#17-7321-82, APC (1.25 uL/1e6 cells)<br>Rat Anti-Mouse IL2 clone JES6-5H4, eBioscience, Cat#48-7021-82, eFluor450 (1.25 uL/1e6 cells)<br>Rat Anti-Mouse IL4 clone 11B11, BioLegend, Cat#504132, PE-Dazzle594 (1.25 uL/1e6 cells)

	Rat Anti-Mouse IL10 clone JES5-16E3, eBioscience, Cat#56-7101-82, AF700 (1.25 uL/1e6 cells)<br>Rat Anti-Mouse CD4 clone GK1.5, BioLegend, Cat#100451, BV605 (2.5 uL/1e6 cells)<br>Rat Anti-Mouse CD8 clone 53-6.7, BD, Cat#563332, BV786 (0.75 uL/1e6 cells)<br>Live/dead stain, eBioscience Cat#65-0866-18, eFluor506 (1:1000)<br><br>Antibodies for ELISpot (all from Mabtech):<br>Anti-monkey IFNgamma, clone 7-B6-1, biotin conjugated (1:1000), Kit catalog #3421M-4APW-10<br>Anti-mouse IFNgamma, clone R4-6A2, biotin conjugated (1:1000), Kit catalog #3321-4APW-10<br>Anti-human IL4, clone IL4-II, biotin conjugated (1:1000), Kit catalog #3410-4APW-10<br><br>Antibodies for IU assay:<br>Rabbit anti-adenovirus, Abcam #Ab6982 (1:8000)<br>Goat anti-Rabbit HRP, Bethyl labs, #A120-101P (1:1000)<br><br>Antibodies for western blot:<br>Mouse anti-Spike S2 monoclonal antibody clone 1A9, GeneTex, Cat#GTX632604 (1:1000)<br>Rabbit Anti-Mouse Actin polyclonal, Bethyl, Cat#A300-485A (1:1000)<br>Goat Anti-Mouse HRP, Bethyl, Cat#A90-116P (1:10,000)<br>Goat anti-Rabbit HRP, Bethyl, Cat#A120-101P (1:10,000)
Validation	All antibodies were validated by the vendor. Validation information can be found on the vendor websites:<br>https://www.thermofisher.com/antibody/product/IFN-gamma-Antibody-clone-XMG1-2-Monoclonal/12-7311-82<br>https://www.thermofisher.com/antibody/product/TNF-alpha-Antibody-clone-MP6-XT22-Monoclonal/17-7321-82<br>https://www.thermofisher.com/antibody/product/IL-2-Antibody-clone-JES6-5H4-Monoclonal/48-7021-82<br>https://www.biolegend.com/nl-nl/products/pe-dazzle-594-anti-mouse-il-4-antibody-10715<br>https://www.thermofisher.com/antibody/product/IL-10-Antibody-clone-JES5-16E3-Monoclonal/56-7101-82<br>https://www.biolegend.com/en-us/products/brilliant-violet-605-anti-mouse-cd4-antibody-10708?GroupID=BLG4745<br>https://www.bdbiosciences.com/en-us/products/reagents/flow-cytometry-reagents/research-reagents/single-color-antibodies-ruo/buv805-rat-anti-mouse-cd8a.612898<br>https://www.thermofisher.com/order/catalog/product/65-0866-14<br>https://www.genetex.com/Product/Detail/SARS-CoV-SARS-CoV-2-COVID-19-spike-antibody-1A9/GTX632604<br>https://www.thermofisher.com/antibody/product/Cytoskeletal-Actin-Antibody-Polyclonal/A300-485A<br>https://www.abcam.com/adenovirus-type-5-antibody-ab6982.html<br><br>PMA/ionomycin and/or SEB stimulated splenocytes were used as a positive control for all cytokine antibodies during panel development.

Eukaryotic cell lines

Policy information about cell lines

Cell line source(s)	HEK-293F (Thermo, #11625019)<br>HEK-293A (Thermo, R70507)<br>HEK293-ES (Expression Systems LLC, #94-007F)<br>Vero-E6 (ATCC, CRL-1586)<br>Vero-E6 (BEI, NR-596)
Authentication	Cell lines were not authenticated
Mycoplasma contamination	All cell lines tested negative for mycoplasma
Commonly misidentified lines<br>(See ICLAC register)	None

Animals and other organisms

Policy information about studies involving animals; ARRIVE guidelines recommended for reporting animal research

Laboratory animals	Mouse: Mus musculus, Balb/cAnNHsd, female, 6 - 8 weeks old (Envigo). Rhesus macaques: Macaca mulatta, Indian-origin, male and female, >2.5 years old (Envigo)
Wild animals	Study did not involve wild animals
Field-collected samples	Study did not involve samples collected from the field
Ethics oversight	Mouse studies were approved by Murigenics IACUC and NHP study was approved by Battelle IACUC

Note that full information on the approval of the study protocol must also be provided in the manuscript.

Flow Cytometry

Plots

Confirm that:

- [x] The axis labels state the marker and fluorochrome used (e.g. CD4-FITC).
- [x] The axis scales are clearly visible. Include numbers along axes only for bottom left plot of group (a 'group' is an analysis of identical markers).
- [x] All plots are contour plots with outliers or pseudocolor plots.
- [x] A numerical value for number of cells or percentage (with statistics) is provided.

Methodology

Sample preparation	Spleens were suspended in RPMI complete (RPMI + 10% FBS) and dissociated using the gentleMACS Dissociator (Milltenyi Biotec). Dissociated cells were filtered using a 40 µm strainer and red blood cells were lysed with ACK lysing buffer (150 mM NH_4Cl, 10 mM $KHCO_3$, 0.1 mM EDTA). Following lysis, cells were filtered with a 30 um strainer and resuspended in RPMI complete. splenocytes were resuspended at a density of 5e6 cells/mL in complete RPMI and following an overnight rest at 4C, 1e6 cells per well were distributed into v bottom 96-well plates. Cells were pelleted and resuspended in 100 uL of complete RPMI containing an overlapping peptide pool containing 316 peptides (each 15 amino acids in length, 11 amino acid overlap) spanning the SARS-CoV-2 spike antigen, at a final concentration of 0.5 µg/mL per peptide (Genscript). A second well with DMSO only was used as a negative control for each sample. After 1 hour of incubation at 37ºC, Brefeldin A (Biolegend) was added to a final concentration of 5 µg/mL and cells were incubated for an additional 4 hours. Following stimulation, cells were washed with PBS and stained with fixable viability dye (eBioscience). Extracellular staining was performed in FACS buffer (PBS + 2% FBS + 2mM EDTA) with the following antibodies: CD4 (GK1.5, Biolegend), CD8 (53-6.7, BD). Cells were then washed, fixed and permeabilized with the eBiosciences Fixation/Permeabilization Solution Kit. Intracellular staining was then performed in permeabilization buffer with the following antibodies: IFNg (XMG21.2, Invitrogen), TNFa (MP6-XT22, eBiosciences), IL2 (JES6-5H4, eBiosciences), IL4 (11B11, Biolegend), IL10 (JES5-16E3, Biolegend).
Instrument	Samples were collected on a Cytoflex LX (Beckman Coulter).
Software	Analysis of flow cytometry data was performed using FlowJo software (version 10.7.1).
Cell population abundance	N/A
Gating strategy	Splenocytes were gated first on lymphocytes (FSC-A vs. SSC-A), then singlets (FSC-A vs FSC-H), then singlets (SSC-A vs SSC-H), then viable (SSC-A vs e506) then CD8+CD4- OR CD4+CD8- (BV786 vs BV605). CD8 and CD4 populations were subsequently gated for each cytokine versus SSC. See Extended Data Fig. 10.

- [x] Tick this box to confirm that a figure exemplifying the gating strategy is provided in the Supplementary Information.
